# Low-level hepatitis B surface antigen by ECLIA stratifies early relapse risk in patients with interferon-based HBV functional cure

**DOI:** 10.3389/fimmu.2026.1864338

**Published:** 2026-07-08

**Authors:** Xiangyong Li, Baoer Wu, Huaping Xie, Hao Hu, Ting Liu, Xu You, Yanhua Bi, Yurong Gu

**Affiliations:** 1 Infectious Department, The Third Affiliated Hospital of Sun Yat-sen University, Guangzhou, Guangdong, China; 2 The Second School of Clinical Medicine, Southern Medical University, Guangzhou, Guangdong, China; 3 Laboratory Department, The Third Affiliated Hospital of Southern medical University, Shenzhen, Guangdong, China; 4 Laboratory Department, The Third Affiliated Hospital of Sun Yat-sen University, Guangzhou, Guangdong, China

**Keywords:** chronic hepatitis B, HBsAg, electrochemiluminescence immunoassay (ECLIA), relapse, functional cure

## Abstract

**Background:**

Current definitions of functional cure in chronic hepatitis B (CHB) rely on conventional assays of HBsAg tested by enzyme-linked immunosorbent assay (ELISA-HBsAg), which may misclassify patients with low-level residual antigenemia as cured, leading to relapse after treatment cessation. This study aims to investigate whether high-sensitivity electrochemiluminescence immunoassay of HBsAg (ECLIA-HBsAg) can provide a much better definition of deep functional cure in CHB patients discontinuing interferon (IFN) add-on therapy.

**Methods:**

This retrospective study included 292 CHB patients reaching interferon-induced functional cure with HBsAg tested by ELISA and conducted a 48-week follow-up. Clinical-virological data were collected at treatment cessation and during follow-up. HBsAg were measured by ELISA and ECLIA, simultaneously. LASSO-Cox regression was used for predictor selection. Model performance was evaluated using time-dependent ROC curves, calibration analysis, and decision curve analysis (DCA). The optimal cutoff was determined via maximally selected rank statistics.

**Results:**

During a 48-week follow-up, 24 patients (8.2%) in total relapsed. Cumulative relapse rates at 12, 24, 36, and 48 weeks were 1.4%, 3.8%, 6.2%, and 8.2%, respectively. ECLIA-HBsAg emerged as the sole independent predictor (HR: 9.32, 95% CI: 4.97–17.47, p<0.001), with an optimal cutoff of 0.38 COI. Patients with ECLIA-HBsAg >0.38 COI exhibited a significantly higher relapse risk (p=0.0029). The model demonstrated strong discrimination (C-index: 0.805; AUCs at 24/36/48 weeks: 0.790, 0.789, and 0.768, respectively) and high clinical utility.The mean lead-time gain of ECLIA-HBsAg over ELISA-HBsAg was 28 weeks in functional relapsers (95% CI: 20.5-35.5 weeks, p < 0.001).

**Conclusions:**

Patients who experience relapse under conventional ELISA definitions often harbor residual HBsAg detectable by high-sensitivity testing. Based on an exploratory cutoff of 0.38 COI, we propose that highly sensitive ECLIA might be incorporated into clinical practice as a new, more stringent standard for defining “deep functional cure”.

## Introduction

Chronic hepatitis B (CHB) remains a major global health challenge, affecting approximately 296 million people worldwide, with a disproportionately high burden in the Asia-Pacific region and China, which together account for nearly one-third of all cases (1, 2). Persistent HBV infection is a leading cause of cirrhosis and hepatocellular carcinoma (HCC), imposing substantial morbidity, mortality, and healthcare burden (3, 4).

The current consensus therapeutic goal is “functional cure,” defined as sustained Hepatitis B surface antigen (HBsAg) loss, with or without anti-HBs seroconversion, together with undetectable HBV DNA off-treatment (5, 6). In clinical practice, this endpoint is typically determined by standard enzyme-linked immunosorbent assays (ELISA), which classify a patient as “functionally cured” when HBsAg levels fall below 0.05 IU/mL (4, 7, 8). However, a considerable proportion of patients who meet these criteria and discontinue therapy subsequently experience relapse, highlighting the inability of standard assays to detect residual antigenemia due to incomplete eradication of viral reservoirs (such as cccDNA), and the need for a much better, more sensitive and suitable method to define “deep (true) functional cure” as end point (9).

Previous post-cessation prediction models have identified several relapse-associated factors, including age, baseline HBV DNA, baseline HBsAg, and consolidation treatment duration (9). Nevertheless, current risk stratification still relies heavily on conventional biomarkers with limited sensitivity near the lower limit of quantification (8). In particular, ELISA-based HBsAg is both a key variable in existing models and a principal criterion for treatment discontinuation decisions, yet it may miss low-level residual antigenemia in patients deemed functionally cured. This sensitivity gap may lead to improper estimation of functional cure and suboptimal follow-up strategies.These clinical relapses highlight a critical limitation: the absolute definition of functional cure—namely, HBsAg loss—is fundamentally dependent on the sensitivity of the HBsAg assay used. Thus, defining “deep functional cure” based on standard ELISA definitions may be clinically insufficient if high-sensitivity testing already detects residual HBsAg.

High-sensitivity assays such as electrochemiluminescence immunoassay (ECLIA) provide a broader dynamic range and can detect HBsAg levels below the ELISA detection threshold, however, it remains insufficiently defined for evaluating “deep functional cure” in clinical practice. In this study, we systematically collected clinical-virological factors in our database of fuctionally cured CHB patients discontinuing interferon (IFN) add-on therapy, and aimed to identify which factors are most strongly associated with “relapse”, or “non-response” in actual, after treatment cessation, particularly evaluating whether HBsAg detected by high-sensitivity ECLIA can be utilized as a more proper factor to refine the criteria for “deep functional cure” as a new clinical endpoint in CHB, thereby refining criteria for safer treatment discontinuation and personalized follow-up.

## Materials and methods

### Study design and patients

This retrospective cohort study was conducted based on the electronic medical record (EMR) system of The Third Affiliated Hospital of Sun Yat-sen University. We consecutively enrolled all adult, non-cirrhotic patients who met the criteria for “functional cure” of chronic hepatitis B and discontinued antiviral therapy between October 2018 and January 2025.

The inclusion criteria were as follows: (1) age ≥ 18 years; (2) patients receiving ongoing nucleos(t)ide analogue (NA) therapy who subsequently received add-on pegylated interferon alfa-2b (Peg-IFN α-2b) combination therapy after their baseline HBsAg levels declined to <1500 IU/mL; (3)achievement of an end-of-treatment (EOT) functional cure (strictly defined as HBsAg loss, with or without anti-HBs seroconversion, together with undetectable HBV DNA) following interferon-based therapy; and (4) a clear record of treatment discontinuation at the time of inclusion, with all patients being strictly off-treatment during the subsequent follow-up period. The primary exclusion criterion was a significant lack of critical baseline data at the time of treatment cessation.

The study protocol was approved by the Institutional Review Board (IRB) of [The Third Affiliated Hospital of Sun Yat-sen University] (Ethics Approval No:[2018]02-218-02). Due to the retrospective nature of the study, the requirement for individual patient informed consent was waived.

### Data collection and endpoint definition

**Figure 1:**
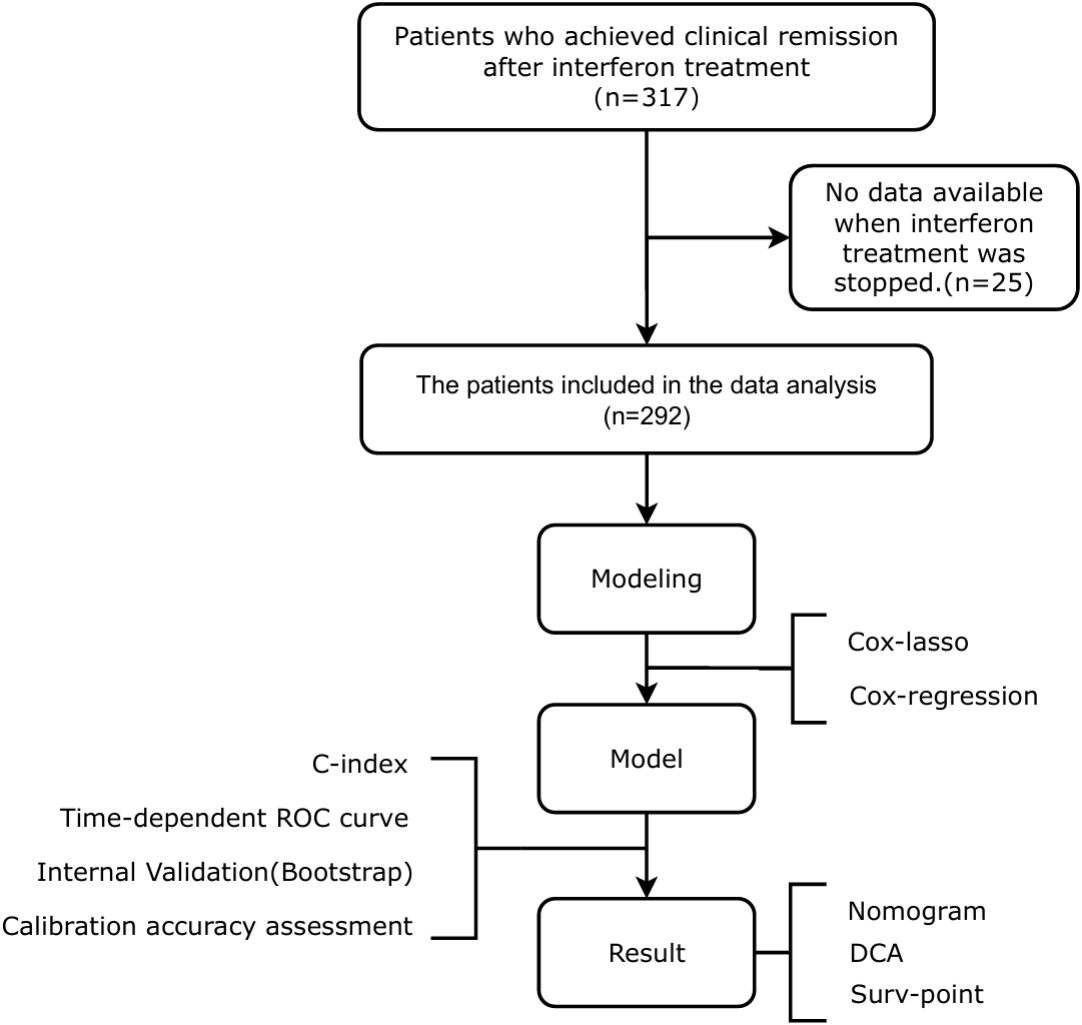
Study design and workflow of the study.

The study design and workflow are illustrated in **Figure 1**. HBsAg levels were measured using two different assays simultaneously. The enzyme-linked immunosorbent assay (ELISA) was performed with commercial kits from Abbott Laboratories (Chicago, IL, USA), and expressed in IU/mL, and the electrochemiluminescence immunoassay (ECLIA) was conducted using reagents from Roche Diagnostics (Mannheim, Germany) and yields a semi-quantitative Cut-off Index (COI) representing the signal-to-cutoff ratio. The primary endpoint of this study was “functional relapse.” In accordance with current clinical guidelines, functional relapse was strictly defined as the reappearance of HBsAg (seroreversion to >0.05 IU/mL), detectable HBV DNA, or both, confirmed by at least two consecutive tests measured at an interval of 4 to 12 weeks during the off-treatment follow-up period (10). Follow-up time was calculated from the date of antiviral therapy discontinuation to the occurrence of a relapse event, death, loss to follow-up, or the end of the observation period, whichever came first.

### Statistical analysis

All statistical analyses were performed using R software (version 4.3.1). A two-sided P-value of less than 0.05 was considered statistically significant.

Given the relatively limited number of relapse events (N = 24), standard multivariable regression including all candidate variables could lead to overfitting. To mitigate this, we employed the Least Absolute Shrinkage and Selection Operator (LASSO) Cox regression to apply a penalty and strictly select the most valuable predictor from 15 candidate variables. The retained variable was then assessed using Cox proportional hazards regression.The Hazard Ratio (HR), 95% Confidence Interval (CI), and P-value were calculated to evaluate the effect size of the variable as an independent risk factor.

### Model performance evaluation

Discrimination: The model’s ability to distinguish functional relapse was assessed using Harrell’s C-index (Concordance index) and Receiver Operating Characteristic (ROC) curves at 24, 36, and 48 weeks.

Calibration: A calibration curve was plotted to evaluate the agreement between the model’s predicted probabilities and the actually observed outcomes.

Internal Validation of the Model: To assess the model’s stability and generalizability, we performed internal validation using the bootstrap resampling method with 1000 repetitions.

Optimal Cutoff Determination: The maximally selected rank statistics method was employed to determine the optimal cutoff value of ECLIA-HBsAg for risk stratification.

Risk Grouping and Survival Analysis: Based on the optimal cutoff, Kaplan-Meier survival curves were generated, and the log-rank test was used to compare the cumulative relapse rates between the two groups.

Nomogram Construction: A visualized nomogram was constructed based on the final Cox model to facilitate the rapid prediction of relapse probability based on a patient’s HBsAg level.

Clinical Utility Assessment: We conducted a Decision Curve Analysis (DCA) to evaluate the clinical utility of the predictive model. Additionally, informed by the Youden’s index and DCA results, we proposed a three-tiered decision-making framework, including screening, routine intervention, and high-risk intervention.

## Results

### Baseline characteristics of the patients

A total of 292 functionally cured chronic hepatitis B patients who discontinued antiviral therapy were included. Baseline demographic and clinical characteristics, including sex, age, liver enzymes (AST, ALT), bilirubin, platelets, and total duration of interferon therapy, were demonstrated in **Table 1**. All patients were HBsAg-negative (<0.05 IU/mL) by standard ELISA at baseline.

**Table 1:** Baseline characteristics of patients in the relapse and non-relapse groups.

Characteristic	CHB patients with functional cure (n=292)
Sex, n (%)	
Female	72 (24.7%)
Male	220 (75.3%)
Age, years	40.00 [34.00, 46.00]
ELISA-HBsAg IU/mL	< 0.05(292, 100%)
ECLIA-HBsAg, COI	0.42 [0.37, 0.51]
HBsAb, IU/L	49.85 [4.09, 255.00]
AST, U/L	40.00 [31.00, 68.25]
ALT, U/L	47.50 [30.75, 78.50]
S/L ratio	0.90 [0.74, 1.10]
Total bilirubin, μmol/L	9.65 [7.70, 11.70]
Platelet count, ×10^9^/L	126.00 [108.00, 166.00]
Injection duration, weeks	48.00 [36.00, 60.00]
Consolidation duration, weeks	22.00 [12.00, 24.00]

*Data are presented as n (%), mean ± standard deviation (SD), or median (interquartile range, IQR).

*CHB, chronic hepatitis B; ELISA, enzyme-linked immunosorbent assay; HBsAg, hepatitis B surface antigen; ECLIA, electrochemiluminescence immunoassay; COI, cutoff index; HBsAb, hepatitis B surface antibody; AST, aspartate aminotransferase; ALT, alanine aminotransferase; S/L ratio, AST/ALT ratio; IQR, interquartile range; SD, standard deviation.

^*^ The lower limit of detection (LOD) for the standard quantitative ELISA HBsAg assay was 0.05 IU/mL. All enrolled patients had HBsAg levels below this threshold at baseline, confirming their functional cure status.

### Relapse events and the clinical-virological characteristics between the patients with relapse and without relapse

During the 48-week follow-up, 24 patients (8.2%) experienced functional relapse, while 268 (91.8%) maintained a sustained response. The median time to relapse was 36 weeks (IQR: 24–42), with cumulative relapse rates of 1.4%, 3.8%, 6.2%, and 8.2% at weeks 12, 24, 36, and 48, respectively.

**Figure 2:**
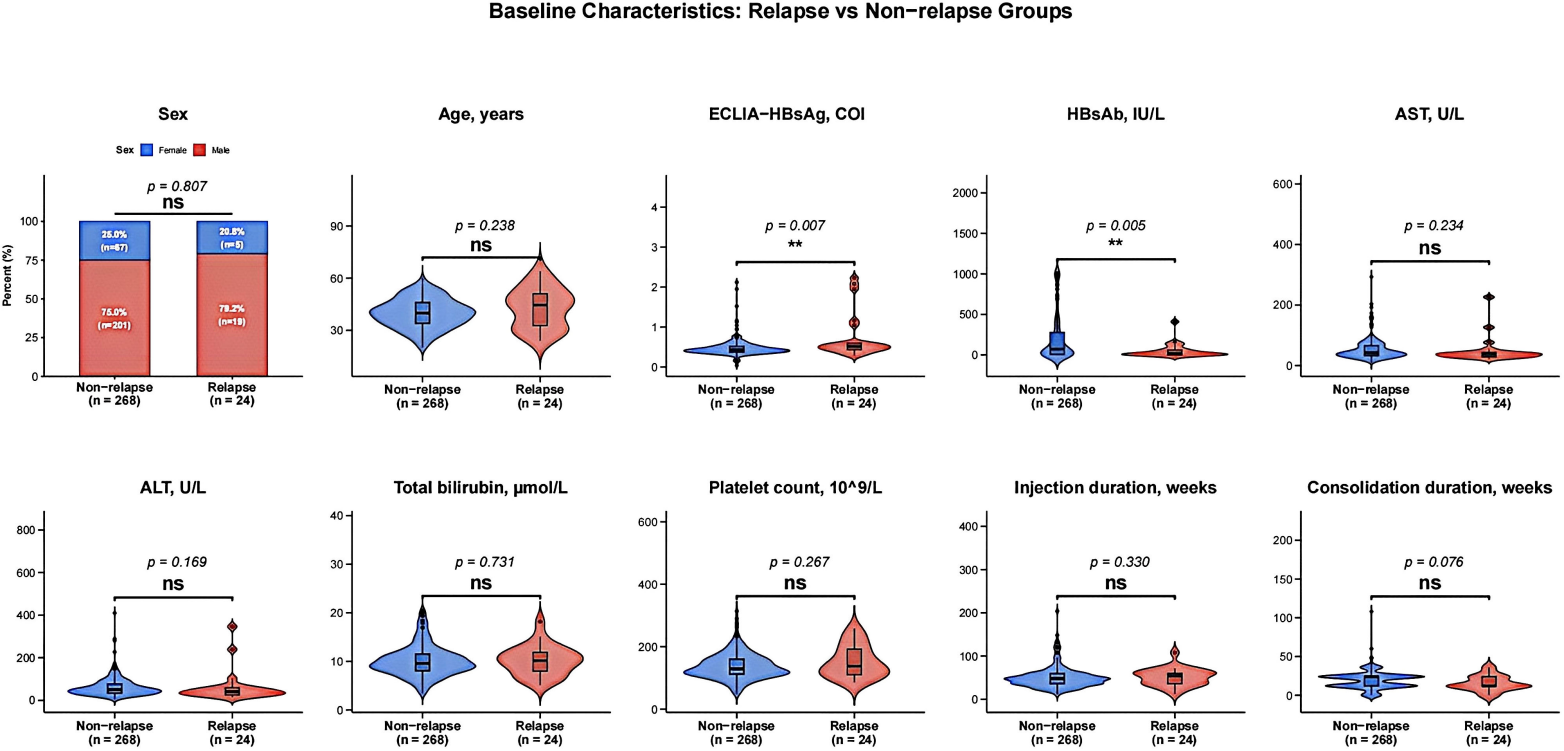
Comparison of baseline characteristics between relapse and non-relapse groups in functionally cured chronic hepatitis B patients, including sex distribution **(A)**, age **(B)**, ECLIA-HBsAg levels **(C)**, HBsAb titers **(D)**, liver enzymes (AST **[E]** and ALT **[F]**), total bilirubin **(G)**, platelet count **(H)**, and interferon therapy duration (injection weeks [I] and consolidation weeks **[J]**). Statistical significance was determined using Mann-Whitney U test for continuous variables and chi-square test for categorical variables. *p < 0.05, **p < 0.01, ***p < 0.001; ns, not significant.

Clinical-virological characteristics were compared between patients with relapse and without relapse. Although HBsAg of ELISA results were below 0.05 IU/ml in both groups, a striking disparity was observed in baseline ECLIA-HBsAg levels: relapsers exhibited significantly higher median levels (Median 0.53 COI; IQR: 0.47–0.82) compared to non-relapsers (Median 0.42 COI; IQR: 0.37–0.48, p < 0.001). As the data exhibited a skewed distribution, median levels were analyzed, revealing that non-relapsers had significantly higher baseline HBsAb compared to patients with relapse [Median 68.45 (IQR: 5.24–277.00) vs. Median 16.10 (IQR: 1.00–60.00) IU/L, p = 0.005]. However, other baseline demographic and clinical characteristics between the two groups, including sex, age, liver enzymes (AST, ALT), bilirubin, platelets, and total duration of interferon therapy, were not different (all the p value >0.05) (**Figure 2**).

### Regression analysis for relapse risk

To identify the factors associated with functional relapse, we analyzed the data with univariable Cox regression and multivariable Cox regression (**Table 2**). In univariable Cox regression, baseline ECLIA-HBsAg was significantly associated with increased relapse risk (HR 1.82, 95% CI 1.48–2.25; p<0.001). Baseline HBsAb was inversely associated with relapse in univariable analysis (HR 0.24, 95% CI 0.07–0.84; p=0.025). Age showed a borderline association (HR 1.46, 95% CI 0.96–2.18; p=0.075), whereas baseline ALT, the total weeks of Peg-IFN therapy, and the consolidation weeks of Peg-IFN therapy were not significant (all p>0.05).

**Table 2:** Univariable and multivariable Cox proportional hazards analyses for functional relapse.

Variable	Univariate		Multivariate	
	HR (95%CI)	P-value	HR (95%CI)	P-value
ECLIA-HBsAg (z-score)	1.82 (1.48–2.25)	<0.001	1.60 (1.28–2.01)	<0.001
Age (z-score)	1.45 (0.96–2.18)	0.075	1.38 (0.83–2.28)	0.217
Baseline HBsAb (z-score)	0.24 (0.07–0.84)	0.025	0.26 (0.05–1.40)	0.117
Sex (Male vs Female)	NA	—	—	—
Baseline ALT (z-score)	0.98 (0.62–1.53)	0.917	—	—
Total injection weeks of Peg-IFN therapy(z-score)	1.09 (0.78–1.52)	0.633	—	—
Consolidation injection weeks of Peg-IFN therapy (z-score)	0.77 (0.49–1.22)	0.263	—	—

^*^ HR, hazard ratio; CI, confidence interval; ALT, alanine aminotransferase.

^*^ Multivariable estimates were derived from a parsimonious model including ECLIA-HBsAg, age, and baseline HBsAb, with multiple imputation (m=5).

Because only 24 relapse events occurred, a parsimonious multivariable model was prespecified to reduce overfitting, retaining ECLIA-HBsAg, age, and baseline HBsAb. In this adjusted model, ECLIA-HBsAg remained an independent predictor of relapse (HR 1.60, 95% CI 1.28–2.01; p<0.001), while age (HR 1.38, 95% CI 0.83–2.28; p=0.217) and baseline HBsAb (HR 0.26, 95% CI 0.05–1.40; p=0.117) were not statistically significant.

Sex was non-estimable in univariable Cox analysis due to sparse-event instability; therefore, it was not included in the final parsimonious multivariable model.As a sensitivity analysis for sex (non-estimable in univariable Cox due to sparse-event instability), we performed Fisher’s exact test on relapse status. No significant association was observed between sex and relapse (male: 19/220 [8.6%] vs female: 5/72[6.9%]; OR 0.79, 95% CI 0.22–2.30; p = 0.807). This result supports the decision not to include sex in the final parsimonious multivariable Cox model.

### LASSO-Cox feature selection and model performance

**Figure 3:**
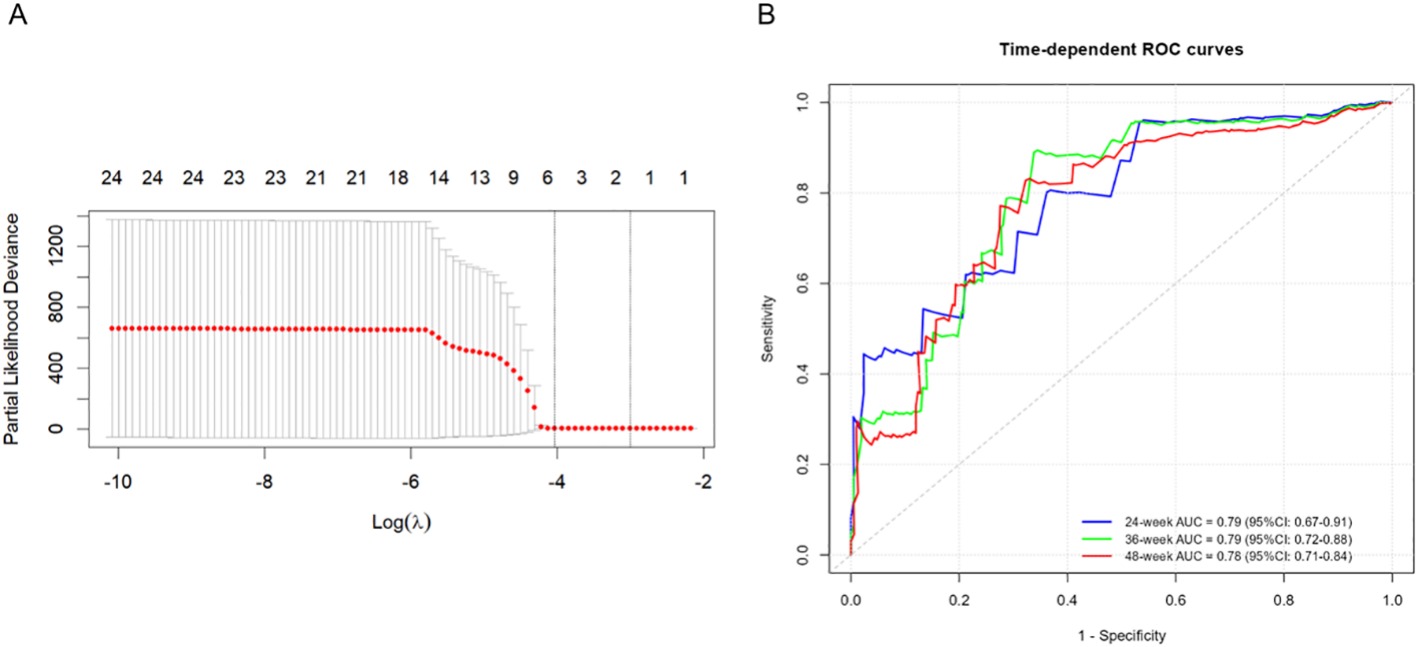
LASSO-Cox variable selection and time-dependent ROC validation. **(A)** LASSO-Cox regression cross-validation and variable selection. As Log(λ) increases, fewer variables are retained. Ultimately, only ECLIA-HBsAg was selected as the predictor. **(B)** Time-dependent ROC curves demonstrating the predictive performance of ECLIA-HBsAg at 24, 36, and 48 weeks, with corresponding Area Under the Curve (AUC) values and 95% confidence intervals estimated via 500 bootstrap resamples.

We then performed LASSO-Cox regression to screen 15 potential clinical and virological variables (**Figure 3A**). As Log(λ) increases, fewer variables are retained, and the model becomes more parsimonious. Only ECLIA-HBsAg was retained, while demographic factors (age, sex) and conventional liver function markers were shrunk to zero coefficients, indicating limited predictive contribution in this cohort.

In the subsequent Cox proportional hazards analysis, ECLIA-HBsAg emerged as the sole independent predictor of relapse (HR 9.32, 95% CI 4.97–17.47; p < 0.001), confirming that residual antigenemia quantified by high-sensitivity ECLIA-HBsAg is strongly associated with the risk of relapse.

Model discrimination and calibration were further evaluated. Time-dependent ROC analysis showed stable AUCs of 0.79 (95% CI: 0.67–0.91), 0.79 (95% CI: 0.72–0.88), and 0.78 (95% CI: 0.71–0.84) at 24, 36, and 48 weeks, respectively (**Figure 3B**). 

### Nomogram-based clinical application and risk stratification

**Figure 4:**
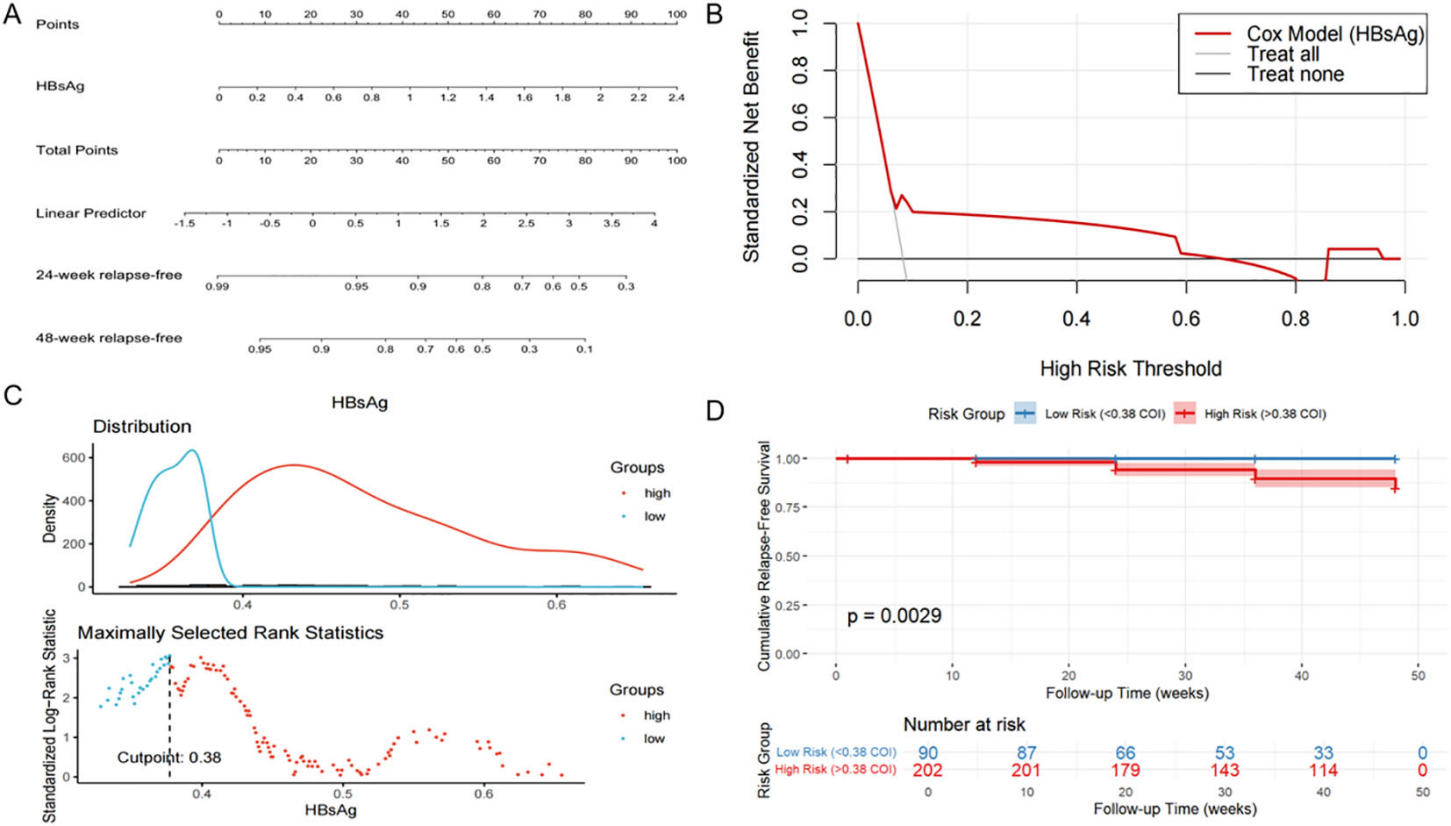
Nomogram-based clinical application and risk stratification **(A)** Nomogram for predicting relapse risk based on ECLIA-HBsAg level at 24 and 48 weeks based on HBsAg levels. **(B)** Decision curve analysis (DCA) of the Cox model based on ECLIA-HBsAg. In the context of our study, “Treat all” assumes all patients receive intensified clinical interventions (e.g., high-frequency monitoring or preemptive resumption of antiviral therapy) regardless of their actual relapse risk, with net benefit decreasing as the threshold probability increases due to rising false-positive costs. “Treat none” assumes no intervention is applied to any patient, resulting in a net benefit of zero across all threshold probabilities. **(C)** Distribution of ECLIA-HBsAg levels and the determination of the optimal cutoff value using maximally selected rank statistics. **(D)** Kaplan-Meier curves for relapse-free survival stratified by ECLIA-HBsAg level (cutoff = 0.38 COI, p = 0.0029).

To facilitate intuitive clinical application, we constructed a visual nomogram based on the single independent predictor, ECLIA-HBsAg, to estimate relapse-free probability at 24 and 48 weeks (**Figure 4A**). Decision curve analysis (DCA) of the Cox model based on HBsAg was explored. It was shown that the Cox model provides greater net benefit than “treat-all” or “treat-none” strategies across a wide range of threshold probabilities, indicating its clinical utility for guiding relapse risk-based interventions. (**Figure 4B**).

To determine the optimal HBsAg cutoff value for risk stratification, we analyzed the distribution of HBsAg levels using maximally selected rank statistics, identifying 0.38 COI as the optimal cutoff (**Figure 4C**). Based on this threshold, patients were stratified into a low-risk group for relapse (ECLIA-HBsAg ≤ 0.38 COI) and a high-risk group for relapse (ECLIA-HBsAg > 0.38 COI). Kaplan-Meier analysis showed clear separation of relapse-free survival between high- and low-risk groups for relapse (log-rank p = 0.0029; **Figure 4D**). The low-risk group achieved near-complete relapse-free survival (>98%).

### Longitudinal Kinetics of HBsAg tested by different methods

**Figure 5:**
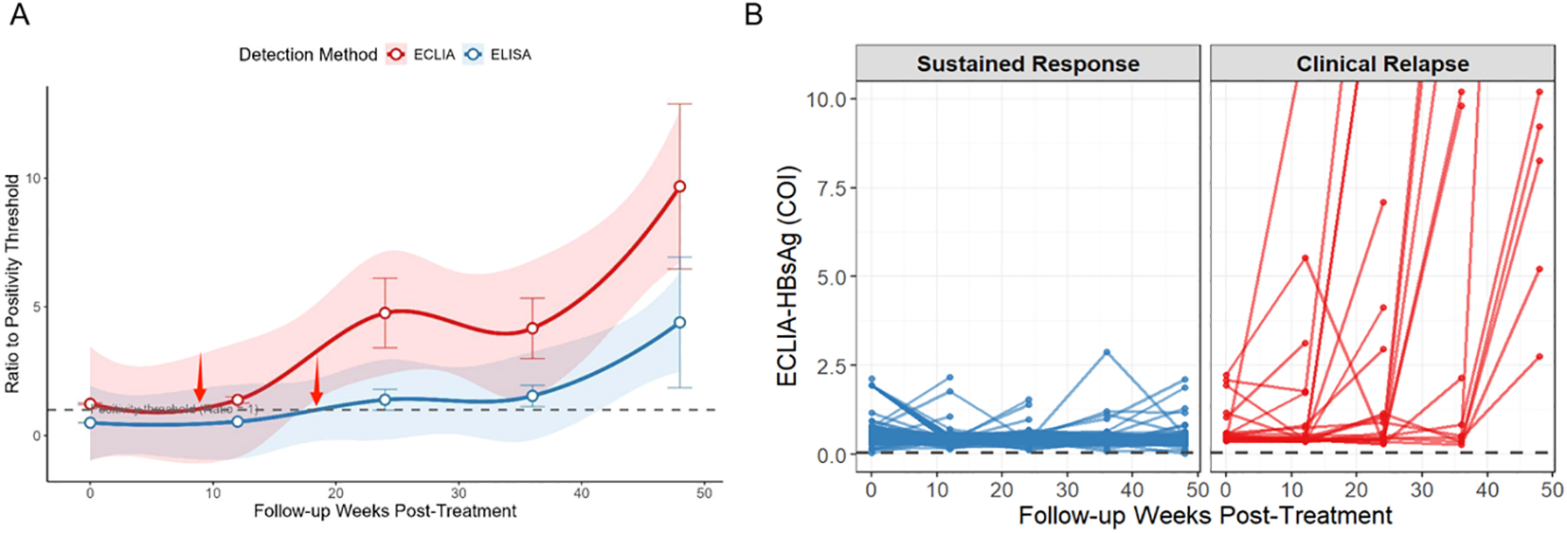
ECLIA-Based HBsAg Monitoring Outperforms Conventional ELISA for Early Prediction of Virological Relapse. **(A)** Normalized longitudinal trajectories of ECLIA-HBsAg and ELISA-HBsAg prior to functional relapse. Measurement values are scaled as ratios to their respective positivity thresholds (dashed line, Ratio = 1). **(B)** Individual longitudinal trajectories of ECLIA-HBsAg stratified by clinical outcome.

Considering ECLIA-HBsAg was found as the very important factor for functional relapse, we visualized the dynamic divergence and longitudinal trajectories of ECLIA-HBsAg levels between groups totally and individually. From an overall perspective of the relapsers, to fairly compare the two assays with disparate units, we visualized their longitudinal trajectories by normalizing the absolute values to their respective positivity thresholds (0.38 COI for ECLIA and 0.05 IU/mL for ELISA). As shown in **Figure 5A**, the normalized ECLIA-HBsAg curve exhibited a continuous and progressive increase from baseline, crossing the positivity threshold (Ratio = 1) at a much earlier stage. In contrast, the ELISA-HBsAg curve remained deeply suppressed for a considerably longer period, only briefly and abruptly crossing its detection threshold prior to the clinical confirmation of relapse. This normalized temporal divergence visually corroborates that ECLIA-HBsAg captures the gradual re-accumulation of low-level antigenemia long before a true relapse is signaled by conventional ELISA(**Figure 5A**).

Meanwhile, the spaghetti plots revealed distinct kinetic patterns of ECLIA-HBsAg individually: while the vast majority of non-relapsers maintained a state of virological quiescence with ECLIA-HBsAg levels fluctuating at extremely low levels (mostly < 1.0 COI), patients who ultimately experienced functional relapse exhibited a clear, progressive upward trend in ECLIA-HBsAg signals. This divergence illustrates the higher sensitivity of ECLIA-HBsAg in tracking low-level antigenemia before conventional ELISA-based positivity (**Figure 5B**).

### Superior early-warning capability of ECLIA-HBsAg over ELISA-HBsAg

To quantify the “lead time” advantage of ECLIA-HBsAg over the standard ELISA-HBsAg in capturing relapse events, we performed a paired comparison of the time-to-detection based on their respective thresholds (0.38 COI for ECLIA and 0.05 IU/mL for ELISA) for each relapsed patient.

**Figure 6:**
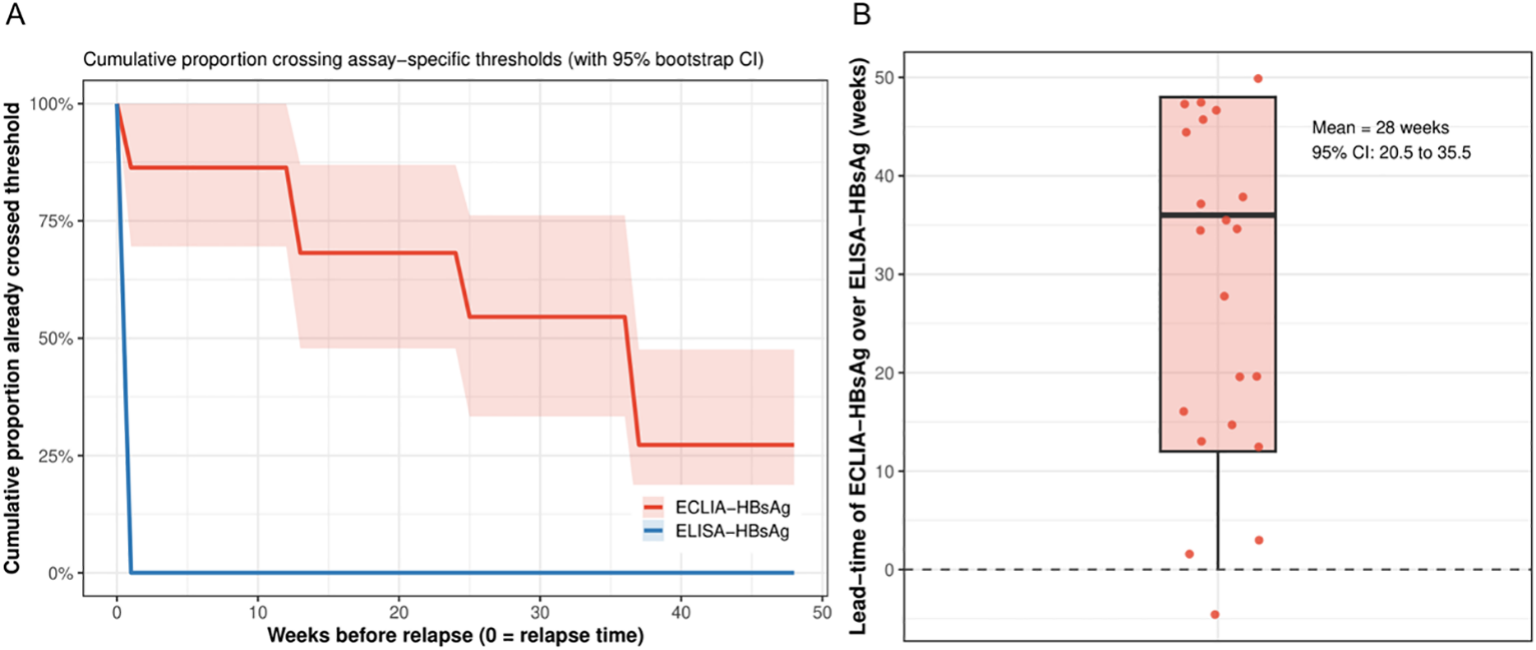
Dynamic early-warning capability and lead-time gain of ECLIA-HBsAg compared with ELISA. **(A)** Dynamic early-warning curves in relapsers. **(B)** Distribution of lead-time gain between ECLIA-HBsAg and ELISA-HBsAg. The mean lead-time gain of ECLIA over ELISA was 28 weeks (95% CI: 20.5–35.5, p < 0.001).

As illustrated in **Figure 6A**, the predictive windows of the two assays differed substantially. The ELISA-HBsAg trajectory showed virtually no early warning capacity, with detection rates rising only at Week 0 and down to none during the follow up. In contrast, the ECLIA-HBsAg trajectory demonstrated a stepwise early stratification capability. Notably, 24 weeks prior to the standard confirmation of relapse, ECLIA-HBsAg had already identified over 50% of the eventual relapsers as high-risk (crossing the proposed 0.38 COI cutoff). By 12 weeks prior to relapse, this proportion increased to approximately 70%.

In a patient-level paired analysis, ECLIA-HBsAg crossed its optimized threshold earlier than ELISA-HBsAg in 100% of the relapse cases. The distribution of lead-time gain is shown in **Figure 6B**. ECLIA-HBsAg demonstrated a median lead time of 36 weeks. The mean lead-time gain of ECLIA-HBsAg over ELISA-HBsAg was 28.0 weeks (95% CI: 20.5–35.5 weeks, p < 0.001).

## Discussion

This study provides novel evidence that residual HBsAg levels, detectable only by electrochemiluminescence immunoassay (ECLIA), complement the traditional definition of “functional cure” based on standard ELISA criteria. While previous models relied on baseline characteristics or less sensitive assays (9, 11, 12), our findings highlight the existence of a “gray zone”—where persistent low-level antigenemia remains below the detection limit of conventional ELISA (0.05 IU/mL). Crucially, our results logically demonstrate that predicting “non-response” or “relapse” in these patients is fundamentally a question of assay sensitivity; patients who ultimately experienced viral rebound actually possessed residual HBsAg and thus would have already been defined as non-responders if assessed by ECLIA at treatment cessation. Consequently, our findings strongly propose that the ECLIA test should be adopted as a much better, more sensitive, and highly suitable endpoint to define “deep functional cure”. In this context, our predictive model and regression analyses function not merely to anticipate relapse, but as an exploratory mathematical framework to identify a data-driven threshold (0.38 COI) that may better distinguish true responders from those who are assay-negative but incompletely cleared. By identifying an exploratory cutoff of 0.38 COI, we demonstrate that patients with sub-threshold antigenemia face a significantly higher risk of relapse, challenging the binary concept of “cure” based solely on current clinical standards.

The biological source of this “gray zone” HBsAg warrants careful consideration. During the natural course of CHB infection, the synthesis of HBsAg is sustained by two distinct templates: the episomal cccDNA and viral sequences that have integrated into the host’s chromosomal DNA (13). Following the achievement of functional cure via interferon-based regimens, the transcriptional machinery of cccDNA is presumed to be largely silenced. Consequently, although our study did not directly measure integrated HBV DNA or cccDNA activity, we hypothesize that the trace HBsAg detected by ECLIA in our cohort (0.05-0.38 COI range) might originate predominantly from integrated HBV DNA rather than active cccDNA replication (10, 13, 14). This potential distinction is crucial: unlike cccDNA-driven antigenemia which fuels robust viral rebound, integration-derived HBsAg could represent a more inert form of residual burden. This mechanistic hypothesis aligns with our clinical observation that, although ECLIA-positive patients had a higher hazard ratio for relapse (HR 9.32), the absolute cumulative relapse rate remained relatively low (8.2%) compared to untreated cohorts. Thus, ECLIA-positivity in this setting could potentially reflect a “leaky” integration reservoir that suggests incomplete immunological control rather than imminent virological failure, necessitating vigilance rather than immediate retreatment.

Furthermore, the persistence of trace HBsAg levels may have profound immunological implications beyond simple viral kinetics. Emerging evidence indicates that the continuous presence of even trace antigen quantities may suffice to perpetuate an immunotolerant environment or sustain T-cell exhaustion, thereby impeding the restoration of effective host surveillance (15). Notably, research published in 2023 demonstrated that the functional recovery of HBsAg-specific B-cell immunity is significantly more pronounced in individuals achieving undetectable HBsAg by high-sensitivity assays than in those retaining low-level residual antigenemia (16). Therefore, the “gray zone” HBsAg detected in our relapsers might act as a “tolerogen,” continuously dampening the host’s surveillance capability. This hypothesis explains why patients with ECLIA-positive results failed to maintain remission, as their immune system was likely never fully “released” from chronic antigenic stimulation, making them vulnerable to viral rebound once the suppressive pressure of interferon was removed.

ECLIA, as an electrochemiluminescence immunoassay, offers enhanced discrimination at low signal intensities (17). Consequently, upon the re-emergence of extremely low levels of HBsAg, it may initially manifest as a sustained increase in the COI. In contrast, conventional ELISA is more susceptible to a “floor effect” near its lower limit of detection. In this scenario, although the true antigen signal is slowly increasing, the measured readings remain within the negative range for an extended period, only crossing the threshold of approximately 0.05 IU/mL after further accumulation of the antigen burden. In this study, our cohort analysis further demonstrated that, in patients experiencing relapse, ECLIA-HBsAg crosses the early warning threshold significantly earlier than ELISA-HBsAg, providing a substantially longer lead time. In other words, this result supports the clinical interpretation that relapse is not an abrupt event occurring only at the moment of ELISA-detectable HBsAg reversion, but is preceded by a window period characterized by the gradual re-accumulation of low-level antigen—a window that ECLIA-HBsAg is better equipped to capture (10, 18).

Beyond HBsAg, markers such as HBcrAg and HBV DNA have been proposed for risk stratification. Dynamic HBcrAg monitoring after nucleos(t)ide analogue withdrawal predicts subsequent functional relapse and may guide post-treatment surveillance (19). Off-treatment HBV DNA levels—particularly the initial magnitude and persistence of elevation—have also been shown to predict biochemical relapse (20), and HBV DNA measured at week 24 off-treatment can further stratify relapse risk when combined with HBsAg (18). However, these markers typically rise concomitantly with or after the resumption of viral replication. In contrast, ECLIA-HBsAg appears to provide valuable clinical utility by quantifying the pre-existing antigen burden even when viral DNA is undetectable. While serum HBV RNA allows for the specific assessment of cccDNA transcriptional activity (pgRNA), ECLIA-HBsAg captures the comprehensive translation products from both cccDNA and integrated sequences, offering a broader surveillance net. Our longitudinal analysis further confirmed that ECLIA-HBsAg levels began to rise weeks before ELISA positivity, providing a crucial lead time for intervention. This suggests that ECLIA acts as an early warning radar, superior to conventional monitoring strategies that often detect relapse only after it has become clinically manifest.

Our findings strongly resonate with the evolving concept of “Functional Cure Heterogeneity” proposed in recent expert consensus statements (5, 10). HBsAg loss is increasingly recognized not as a single binary event but as a spectrum. A recent multi-center analysis suggested distinguishing between “Assay-negative” (standard ELISA) and “True-negative” (high-sensitivity) states, terming the latter as “Deep Functional Cure” (21). Individuals attaining this deep functionality exhibited a markedly superior durability of response, with lower relapse rates than those categorized as discordant (ELISA-negative but ECLIA-positive). As we advocate for this stricter ECLIA-defined endpoint, predicting which patients can achieve such a profound response becomes paramount. Literature has securely demonstrated the importance of HBsAg quantification prior to therapy as a powerful proxy for immune control, particularly utilizing the universally accepted pre-treatment cut-off of 1000 IU/mL (22). Extrapolating from this foundational evidence, we hypothesize that an initial HBsAg level below 1000 IU/mL before IFN add-on therapy is the best predictor of achieving ECLIA-defined deep cure. Specifically, our study cohort exclusively consists of patients who successfully achieved ELISA-defined functional cure at the end of treatment. When we retrospectively reviewed their data, we found that patients capable of achieving such an endpoint inherently tend to have lower initial HBsAg burdens; approximately 95.9% of patients in our cohort already had pre-treatment HBsAg levels below 1000 IU/mL before initiating IFN add-on therapy. Because of this highly skewed demographic, the statistical distribution in this particular dataset was heavily unbalanced, preventing us from conducting a statistically robust and effectively powered analysis for this baseline variable. Therefore, future large-scale prospective studies with broader pre-treatment demographics are urgently needed to definitively confirm this 1000 IU/mL cut-off for predicting true response.

By proposing a specific cutoff of 0.38 COI, our study provides a practical quantitative metric to define this “Deep Cure” status. However, it is important to emphasize that this specific 0.38 COI threshold was strictly data-driven, derived via maximally selected rank statistics in our specific cohort. Furthermore, while we applied advanced methodologies framework (such as the nomogram and DCA) to robustly evaluate this predictor, utilizing these comprehensive modeling approaches for a single predictor derived from a limited number of relapse events (N = 24) inherently carries a risk of overfitting. This paradigm shift advances the field beyond a binary definition, advocating for a stratified framework in which ECLIA-negativity signifies a superior tier of viral clearance, which could potentially inform more personalized monitoring strategies and identify candidates for less intensive follow-up, pending validation in larger clinical cohorts. From a practical perspective, rather than redefining the established criteria, further stratifying the traditional concept of “functional cure” to include ECLIA negativity (a “deep functional cure”) could potentially optimize long-term management. While ECLIA reagents are marginally more expensive than conventional ELISA in some regions, the cost difference is negligible compared to the financial burden of managing a full-blown functional relapse or severe disease flares leading to hepatic decompensation (23). In resource-limited settings like parts of China, a stratified approach is feasible: routine ELISA can remain the primary screening tool, while ECLIA is reserved as a “confirmatory gatekeeper” for patients preparing to stop therapy or those with ambiguous ELISA results (24). For patients with off-treatment ECLIA-HBsAg > 0.38 COI, we recommend intensified monitoring (e.g., every 1–2 months) rather than standard 3–6 month intervals (25), ensuring that limited healthcare resources are targeted toward those at highest risk.

Several limitations warrant acknowledgment. First, the study design is inherently limited by its retrospective nature and single-center recruitment, involving a relatively restricted cohort size (N = 292) and a low frequency of relapse events(N = 24). Consequently,although baseline HBsAb exhibited differences between groups, its strict predictive value for relapse may be overstated in this limited sample and warrants verification. The identifying cutoff of 0.38 COI should be interpreted as exploratory and data-driven, requiring external validation in larger, multicenter cohorts before broad clinical adoption. Second, we did not perform paired liver biopsies to directly quantify intrahepatic cccDNA or integration events, relying instead on serological surrogates to assess residual antigenemia. Importantly, low-level HBsAg positivity should not be interpreted as direct evidence of active viral replication, as it likely reflects antigen expression from integrated HBV DNA rather than actively transcribing cccDNA. Finally, the cost-effectiveness model discussed above is theoretical and warrants formal health economic evaluation in diverse healthcare systems.

In summary, ECLIA-based HBsAg measurement reveals a hidden burden of residual low-level antigenemia in patients conventionally considered “cured” by ELISA. Rather than serving merely as a tool to predict relapse, the proposed exploratory cutoff of 0.38 COI provides a practical metric to redefine and confirm “deep functional cure”, distinguishing true responders from those with incomplete clearance. Moving forward, incorporating high-sensitivity HBsAg assays as a stringent clinical endpoint represents a precise strategy to evaluate true functional cure.

## Data Availability

The raw data supporting the conclusions of this article will be made available by the authors, without undue reservation.
